# Discord between morphological and phylogenetic species boundaries: incomplete lineage sorting and recombination results in fuzzy species boundaries in an asexual fungal pathogen

**DOI:** 10.1186/1471-2148-14-38

**Published:** 2014-03-03

**Authors:** Jane E Stewart, Lavern W Timmer, Christopher B Lawrence, Barry M Pryor, Tobin L Peever

**Affiliations:** 1 Department of Plant Pathology, Washington State University, Pullman, WA, USA; 2 Citrus Research and Education Center, University of Florida, Lake Alfred, FL, USA; 3 Virginia Bioinformatics Institute, Blacksburg, VA, USA; 4 Division of Plant Pathology and Microbiology, School of Plant Sciences, University of Arizona, Tucson, AZ, USA; 5 Current address: Department of Plant Pathology, University of Georgia, Athens, Georgia

**Keywords:** Coalescent, Species delimitation, Species tree, Gene tree

## Abstract

**Background:**

Traditional morphological and biological species concepts are difficult to apply to closely related, asexual taxa because of the lack of an active sexual phase and paucity of morphological characters. Phylogenetic species concepts such as genealogical concordance phylogenetic species recognition (GCPSR) have been extensively used; however, methods that incorporate gene tree uncertainty into species recognition may more accurately and objectively delineate species. Using a worldwide sample of *Alternaria alternata* sensu lato, causal agent of citrus brown spot, the evolutionary histories of four nuclear loci including an endo-polygalacturonase gene, two anonymous loci, and one microsatellite flanking region were estimated using the coalescent. Species boundaries were estimated using several approaches including those that incorporate uncertainty in gene genealogies when lineage sorting and non-reciprocal monophyly of gene trees is common.

**Results:**

Coalescent analyses revealed three phylogenetic lineages strongly influenced by incomplete lineage sorting and recombination. Divergence of the citrus 2 lineage from the citrus 1 and citrus 3 lineages was supported at most loci. A consensus of species tree estimation methods supported two species of *Alternaria* causing citrus brown spot worldwide. Based on substitution rates at the endo-polygalacturonase locus, divergence of the citrus 2 and the 1 and 3 lineages was estimated to have occurred at least 5, 400 years before present, predating the human-mediated movement of citrus and associated pathogens out of SE Asia.

**Conclusions:**

The number of *Alternaria* species identified as causing brown spot of citrus worldwide using morphological criteria has been overestimated. Little support was found for most of these morphospecies using quantitative species recognition approaches. Correct species delimitation of plant-pathogenic fungi is critical for understanding the evolution of pathogenicity, introductions of pathogens to new areas, and for regulating the movement of pathogens to enforce quarantines. This research shows that multilocus phylogenetic methods that allow for recombination and incomplete lineage sorting can be useful for the quantitative delimitation of asexual species that are morphologically indistinguishable. Two phylogenetic species of *Alternaria* were identified as causing citrus brown spot worldwide. Further research is needed to determine how these species were introduced worldwide, how they differ phenotypically and how these species are maintained.

## Background

The delimitation of species and evolutionary relationships among them is fundamental to biology. However, the application of species concepts to putatively asexual taxa can be difficult and controversial [1,2]. Not all species concepts can be applied to asexual species, and some researchers have even suggested that asexual lineages do not represent species at all [3]. For example, the morphological species concept (MSC) or biological species concept (BSC) may not be adequate for delineating asexual fungi [2,4,5] especially where morphological differences are not observed. However, any new allele conferring an adaptive advantage to an asexual organism in a particular ecological niche may be selected. Selective pressure on that gene is expected to affect the entire genome through genetic hitchhiking thus having the potential to rapidly form a new cryptic species [4]. These discrete entities may be recognized as species rather than as part of continuous distribution of phenotypes. Asexual taxa are also expected to diverge into discrete lineages under processes such as divergent selection and/or geographic isolation [4].

To date, the systematics of asexual fungi has relied heavily on phylogenetic approaches to study cryptic speciation among closely related taxa [5-7]. In asexual fungi, phylogenetic species concepts can identify phylogenetically distinct lineages with the implication that new species have formed that are not yet morphologically distinct [8]. These methods most often involve the concatenation of sequence alignments, using methods such as the genealogical concordance phylogenetic species recognition (GCPSR) which is an operational criterion for species recognition [2,9]. The GCPSR focuses on species identification through multi-gene genealogies and reciprocal monophyly to identify fungal species [5]. This method has applicability for both asexual and sexual lineages and species boundaries are estimated by concordant clades of multi-gene genealogies. The absence of monophyly and conflict among the multiple gene trees identifies species limits for taxa [5]. However, species boundaries of closely related taxa, in the initial stages of divergence, can be difficult to ascertain using multilocus phylogenetic methods because gene trees of recently diverged taxa can differ substantially in their evolutionary histories [10].

Processes such as incomplete lineage sorting, recombination, and horizontal transfer can cause discord among gene and species trees, masking true evolutionary relationships among closely related taxa [11]. Incongruence, in itself, can signal possible recombination, reticulation, and incomplete lineage sorting. Individual gene trees may have different evolutionary histories [12,13] which limits the accuracy of species tree estimation using concatenation of loci [14]. Incomplete lineage sorting is caused when ancestral polymorphisms persist through speciation events and each ancestral polymorphism can lead to different alleles carried among descendants [14,15]. Coalescent-based methods, which stochastically join sampled gene lineages as they are followed back in time, have been developed to incorporate lineage sorting and the presence of incongruent genomic regions into phylogenetic estimation procedures [16-18], even in the presence of lineage sorting and lack of reciprocal monophyly at any single locus [10].

Coalescent methods have recently been used to assess species trees for a range of taxa including sexually reproducing species such as the Tennessee cave salamanders (*Gyrinophilus*; [19]), tropical lowland birds (*Manacus*; [20]), grasshoppers (*Melanoplus*; [10]), and rice [21]. Few researchers, however, have examined the utility of these methods for closely related asexual taxa that are morphologically indistinguishable [22]. Phylogenetic analyses of closely-related taxa, such as rice [21], *Drosophila*[23], and cryptic fungal species, such as *Penicillium*[22,24], are at the intersection of population genetics and phylogenetics where the effects of coalescent stochasticity results in high levels of gene tree incongruence [17,25-27]. Estimating species trees for these taxa can be problematic; these methods could prove to be useful for closely-related asexual fungal taxa.

The putatively asexual citrus pathogen, *Alternaria alternata*, provides an ideal case study for the application of quantitative species recognition using species tree estimation methods that incorporate uncertainty in gene trees. Andrew et al. [28] developed a species phylogeny for small-spored *Alternaria* using four genomic regions including a protein coding gene and three anonymous, non-coding regions. Significant incongruence was found among gene genealogies and several putative recombination events were identified within two of the non-coding regions indicating divergent evolutionary histories among the loci [28]. Hypotheses to explain this incongruence included recombination and incomplete lineage sorting. A recent study of the mating system of *A. alternata* causing brown spot in Florida found signatures of recombination [29] but studies of the larger worldwide population of the pathogen are lacking.

Currently, there is a large discord between the number of morphological and phylogenetic species that are thought to cause citrus brown spot. The fungus infects tangerines and mandarins (*Citrus reticulata* Blanco) and tangerine x grapefruit (*C. reticulata* x *C. paradisi* Macfad.) hybrids worldwide. When first reported in Australia [30], the pathogen was identified as *A. citri* Ellis & N. Pierce due to its morphological similarity to the causal agent of a postharvest disease, citrus black rot. Since this time, the pathogen has been referred to as *A. alternata* ‘tangerine pathotype’ based on morphological similarity to *A. alternata*[31-33]. Further, molecular comparisons were performed using restriction fragment length polymorphisms (RFLPs) of the nuclear ribosomal DNA (internal transcribed spacer region, ITS) among 11 closely related, small-spored *Alternaria* taxa. Results from this study showed that morphologically similar *Alternaria* species collected from different hosts and that produce host specific toxins shared common RFLP fingerprints and identical ITS sequences, leading the authors to conclude that *Alternaria* fungi known to produce host-specific toxins are intraspecific variants of *A. alternata* specialized in host pathogenicity [33].

Ten new *Alternaria* species have been described from citrus hosts [34] and phylogenetic studies have attempted to map these morphospecies onto phylogenies estimated from molecular data. Using a worldwide sample of isolates, Peever et al. [35,36] and Andrew et al. [28] evaluated these morphospecies using phylogenetic criteria and found three distinct lineages (labeled Clades 1, 2 and 3 in [35]). Two of these lineages were found in Florida (Clades 1 and 2), whereas the third lineage occurred only in Turkey, Israel, Australia and South Africa (Clade 3). These three worldwide lineages corresponded to several morphospecies including *A. citriarbusti* (Clade 1), *A. tangelonis* and *A. colombiana* (Clade 2), and *A. dumosa*, *A. turkisafria*, *A. perangusta* and *A. interrupta* (Clade 3) [34]. Peever et al. [36] further tested the concordance between the 10 citrus-associated morphospecies [34] on citrus using a broader range of isolates. Peever et al. [36] found eight distinct *Alternaria* clades from citrus hosts that could be interpreted as phylogenetic species under the GCPSR concept. This incongruence between the number of species defined using morphological and phylogenetic criteria raises significant questions about the number of *Alternaria *taxa that cause brown spot disease.

Coalescent analyses of gene geneaolgies, which describes descendent/ancestor relationships where the gene of interest undergoes coalescence to a common ancestor, can be used to examine the evolutionary history of a gene backwards in time and can be used to incorporate incomplete lineage sorting into phylogenetic analyses [37]. Currently, there is a lack of agreement among researchers about how many *Alternaria* species cause citrus brown spot, ranging from as many as 10 species to as little as 1 species. The main objective of this study was to quantitatively estimate the number of species of *Alternaria* causing citrus brown spot on a worldwide scale utilizing newly developed methods that incorporate the coalescent and account for recombination or incomplete lineage sorting. The evolutionary histories and recombination of known genetically distinct lineages, citrus 1, citrus 2 and citrus 3 were evaluated from a larger worldwide sample of *A. alternata* tangerine pathotype and more loci in order to establish species boundaries of these previously observed phylogenetic lineages using gene sequence concatenation methods and several approaches that incorporate uncertainty in gene genealogies when lineage sorting and non-reciprocal monophyly of gene trees is common.

## Methods

### Isolates

**Figure 1:**
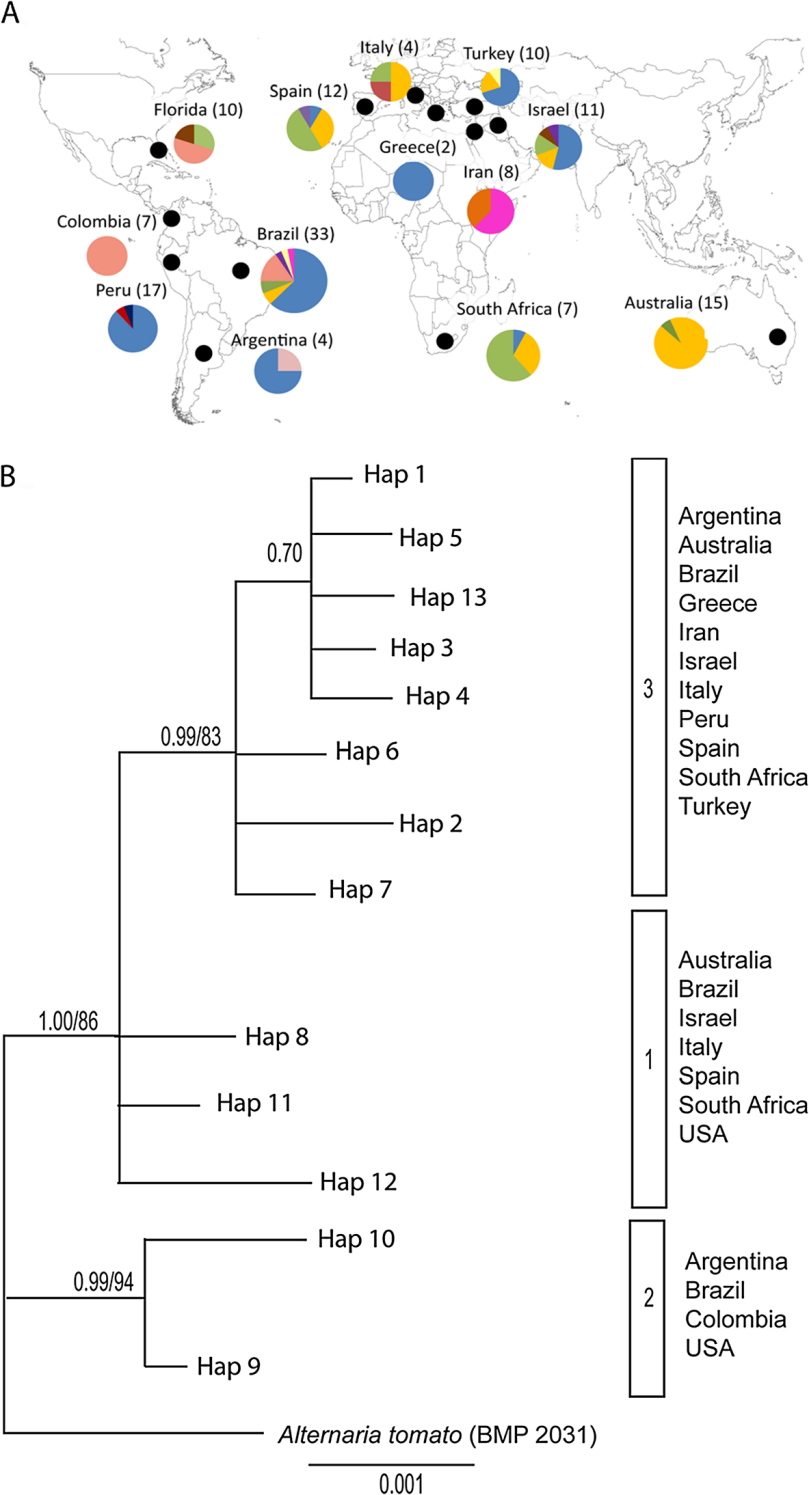
**A. Geographic origin of*Alternaria alternata*sampled from citrus in 12 countries (small black circles).** The numbers of isolates sampled from each country are in parentheses. The pie chart for each country represents the number of *endoPG* haplotypes with each color denoting a different *endoPG* haplotype [(Hap1 (Fuschia), Hap2 (orange), Hap3 (blue), Hap4 (dark blue), Hap5 (red), Hap6 (maroon), Hap7 (yellow), Hap8 (green), Hap9 (pink), Hap10 (light pink), Hap11 (brown), Hap12 (purple), and Hap13 (light yellow)]. **B**. An *endo-polygalacturonase* (*endoPG*) phylogeny estimated among a worldwide sample of citrus brown spot isolates using Bayesian inference with *A. tomato* as an outgroup. Three phylogenetic lineages identified correspond to Clades 1, 2, and 3 of Peever et al. [35]. Node support is given as posterior probabilities and bootstrap values based on Bayesian and likelihood analyses.

One hundred and forty two isolates were collected from brown spot lesions on cultivars of tangerines, mandarins (*Citrus reticulata* Blanco) and tangerine x grapefruit hybrids [*C. reticulata* x *C. paradise* (Macfad.)] in 12 countries, including Argentina (AR), Australia (AU), Brazil (BZ), Colombia (CB), Greece (GR), Iran (IR), Israel (IS), Italy (IT), Peru (PE), Spain (SP), Turkey (TU), and USA (FL). Sixty-five of these isolates from Australia, Colombia, Israel, South Africa, and United States overlapped with a previous study ([35]; Figure 1A, Additional file 1: Table S1). Isolates from Argentina, Australia, Brazil, Colombia, Greece, Iran, Israel, Italy, Peru, Spain, Turkey, and USA were sampled from tangerine hybrids in several geographically separated citrus groves within each country. Australian isolates were collected from tangerine hybrids in the Narara arboretum (Narara, New South Wales), and isolates from Florida, USA were collected from a small area (2,500 m^2^) in a single grove of Minneola tangelo [35,36,38].

### DNA extraction

Fungi were cultivated in potato dextrose broth (FisherSci, Pittsburg, PA) for 5–7 days at room temperature on an orbital shaker at 150 rpm. Genomic DNA was extracted from powdered, lyophilized mycelium following the methods of Peever et al. [38], using either a Qiagen DNeasy Kit or a Phenol-Chloroform procedure. Extracted DNA was quantified using a Nanodrop1000 (NanoDrop products, Wilmington, DE, USA), and a total of 30 ng was used as template for PCR. Isolates were maintained in long-term storage on sterilized filter paper at −20°C as previously described [38].

### Endo-polygalacturonase sequencing

Each isolate was sequenced at the *endo-polygalacturonase* locus [39] using primers and conditions similar to those used by Peever et al. [35,36,40] and Andrew et al. [28]. This region has been extensively used because other commonly used genomic regions, such as ribosomal regions, mitochondrial large and small subunits, and the beta-tubulin gene, show little variation among *Alternaria* isolates collected from citrus [41]. Amplified products were visualized in 2% ethidium bromide-stained agarose gels. Amplified DNA fragments were sequenced directly on both strands following treatment with EXOSAP-IT (USB, Cleveland, OH) using the Big Dye terminator kit (Applied Biosystems, Foster City, CA). Sequence reads were performed on either a PE Biosystems model 3700 automated DNA Sequencer by the Laboratory for Biotechnology and Bioinformatics at Washington State University, Pullman, WA or at Elim Biopharmaceuticals, Inc, Wayward, CA.

### *OPA1-3, OPA2-1,* and *Flank-F3 sequencing*

A subset of isolates with unique *endoPG* haplotypes representing each sampled location (*n* = 34) was selected for additional sequencing and analyses. Additional loci included two anonymous, non-coding SCAR markers OPA1-3 and OPA2-1 [28], and one non-coding microsatellite flanking region Flank-F3. Loci OPA1-3 and OPA2-1 were previously used for phylogenetic studies of small-spored *A. alternata*[36,42]. Amplification and sequencing conditions were as previously reported [28,36,40,42]. Clone sequences containing microsatellites [43] were downloaded from GenBank [accession: DQ272483 to DQ272487], and primers were designed to amplify the microsatellite flanking regions using Primer 3 [44]. Primer sequences for AA-Flank-F3 were (Flank3F-5′-AGCCAAAACACGTTGATACC-3′/ Flank3R-5′ ATCCGCAGCGAAAAGAACT-′3). Twenty microliter PCR reaction mixtures contained 20 ng genomic DNA, 1 × PCR buffer (New England Biolabs (NEB), Ipswich, MA), 4 nmol of each dNTP (NEB), 50 pmol primer, and 1U of Taq polymerase (NEB). Cycling conditions consisted of denaturation at 94°C for 4 min; 44 cycles of 94°C for 1 min, 55°C for 30 sec, and 72°C for 2 min; final extension was at 72°C for 7 min depending on the optimal conditions for each primer set.

### Phylogenetic analyses and congruence among loci

Each locus was analyzed independently. Maximum likelihood and Bayesian phylogenetic analyses were performed for each locus using PhyML [45] and MrBayes 3.0 [46]. DT-ModSel [47] was used to estimate the nucleotide substitution models best representing each dataset. An Kimura K80 model was selected for the *endoPG* (base frequencies = equal; transversion = 5.29; proportion of variable sites = 0), and OPA2-1 (base frequencies = equal; transversion ratio = 5.52; proportion of variable sites = 0). The Kimura K80 model with proportion of invariable sites was selected for OPA1-3 (base frequencies = equal; tratio = 1.47; proportion of variable sites = 0). The Jukes Cantor 69 model was selected for Flank-F3 (base frequencies = equal; proportion of variable sites = 0). Maximum likelihood analyses were performed under the heuristic search with TBR branch-swapping, and bootstrap support was estimated using 1000 pseudoreplicates. For Bayesian analyses, Metropolis-coupled Markov chain Monte Carlo searches included 2 runs with four chains each run for 3,000,000 generations and ensuring that the average split frequencies between the runs was less than 1%. Trees were sampled every 200 generations. Each run generated 60,001 trees of which the first 18,000 trees (30% of the total number of generations) were discarded as “burnin”, as visually determined by evaluating log files in TRACER version 1.5 [48].

In order to test topology congruence among phylogenies from different loci, the Shimodaira-Hasegawa (SH) test of topological congruence [49] was conducted on the Bayesian phylogenies as implemented in PAUP*10_4b [50] with 1000 RELL resampling replicates.

### Coalescent analyses

Ancestral histories of the citrus brown spot lineages were estimated using the coalescent [37,45]. Sequence data were aligned and edited manually by eye and using clustalW implemented in BioEdit v7.0.53 for Windows [51]. Isolates were assigned to haplotypes using DnaSP v 5.1 [52,53]. To verify the suitability of each locus for coalescent analyses, the neutrality of each locus was estimated using Fu and Li’s D and Tajima’s *D* and potential recombination within each locus was examined using Rmin [54] as implemented in DnaSP. Incompatibility matrices [55] were estimated in SNAP Clade and SNAP Matrix as implemented in SNAP workbench [56] to visualize incompatible nucleotide sites, such as those arising from recombination or recurrent mutation. Sequences were collapsed into unique haplotypes using SNAP map [57] and SITES version 1.1 [58] by removing indels and incompatible sites.

Evolutionary histories were simulated using coalescent analyses for each locus. Two loci Flank-3 and OPA1-3 showed evidence for recombination and therefore coalescent analyses for all genomic regions were implemented using recom version 5.8 (within SNAP workbench), which allows for coalescent analyses with recombination, assuming the infinite-sites model, neutral evolution, panmixia and constant population size [59]. Using a haploid coalescent model, recom5.8 estimates population recombination rate, ρ, (2N_e_r) and mutation rate, θ (2N_e_μ). These estimates were then used to obtain estimates of the number of recombination events and the time to the most recent common ancestor (TMRCA). For all analyses, the ancestral state of each segregating site was estimated by comparing each site to that of *A. tomato* isolate BMP2031 [60]. Sites which had ambiguous ancestral states were removed from the datasets. OPA1-3 had five sites (205, 230, 301, 382, and 436) with three states and one site (349) with four states, and the *endoPG* had one site (346) with three states. Coalescent analyses were performed in SNAP workbench [56], with five independent runs (1 million simulations each) per genomic region to ensure convergence for each parameter estimate. Conditions were switched to + b for genomic region OPA1-3, which aborts low probability paths and returns to zero. Gene geneaologies and minimal recombination graphs (ARGs) were constructed to graphically represent the evolutionary history of the citrus lineages estimated by the coalescent. No putative recombination events were detected within *endoPG* and OPA2-1. Genetree [61], which assumes coalescent analyses without recombination, was used to estimate the coalescent gene genealogies for these regions and compared to the recom5.8 results. Five independent simulations with 1 million runs each were conducted on haplotypes of each region to estimate the ages of mutations and the TMRCA, as inferred by an *A. tomato* rooted tree, to assess convergence. A graph of the tree was generated with coalescent unit times using Treepic [62]. An ancestral recombination graph (ARG) can be used to visualize a recombining coalescent history that cannot be displayed using a bifurcating tree [63]. The ancestral history of the recombining OPA1-3 and Flank-F3 haplotypes were reconstructed using a parsimony approach that accounts for both mutation and recombination backwards in time. Beagle [63] was used to produce an ARG for Flank-F3, whereas kwarg was used for OPA1-3 [63]. Beagle computes minimum recombination histories with an exhaustive approach. Kwarg, on the other hand, implements a heuristic search for plausible histories and does not guarantee the minimal recombination history. Beagle was run for both genomic regions, however due to the increased complexity and putative number of recombination events within OPA1-3, each trial run crashed.

### Species tree estimation

Four methods were used to estimate species trees among the four gene trees. These included concatenation, genealogical concordance phylogenetic species recognition (GCPSR; [5]), minimizing deep coalescence (MDC; [14,17,64]), and a mixture of coalescent and the Yule process (*BEAST) [48]. For MDC and *BEAST, taxa are required to be assigned to species *a priori*. Therefore, taxa were assigned to species based on identified *endoPG* clades (citrus 1, citrus 2, and citrus 3, Additional file 1: Table S1).

A phylogeny of the concatenated dataset was implemented in MrBayes. Loci were partitioned and the previously estimated evolutionary models were used for Bayesian analyses. Metropolis-coupled Markov chain Monte Carlo searches included 2 runs with four chains each run for 3,000,000 generations and ensuring that the average split frequencies between the runs was less than 1%. Each run generated 60,001 trees of which the first 18,000 trees (30% of the total number of generations) were discarded as “burnin”.

GCPSR identifies species boundaries by comparing multiple gene trees among the same set of taxa [5,65]. Putative species are identified when representatives of a species formed well-supported clades (95 posterior probability/70 bootstrap) in all gene trees [66]. Bayesian and likelihood tree searches were used to estimate species within the citrus brown spot worldwide population according to GCPSR criteria.

The MDC approach assumes that discordance of gene trees is the result of incomplete lineage sorting [14,17]. The deep coalescence measure is a count of the number of extra gene lineages that result from fitting a gene tree into a species tree, thereby summing the extra gene lineages as a measure of discordance. MDC then searches for a species tree by minimizing the number of deep coalescences across loci. MDC analysis was implemented in Mesquite v2.73 [67]. Gene tree uncertainty was accommodated into species tree inference by resampling (500 times) the posterior probability of tree topologies obtained from the Bayesian phylogenetic analyses using the Mesquite software module AUGIST [64]. To fit the gene trees into a species tree that minimized the number of deep coalescence across loci, gene trees were considered rooted (*A. tomato* as the root) and a heuristic search utilizing subtree pruning and re-grafting was used. All equally parsimonious species trees were retained, and a 50% majority-rule consensus tree was generated. Biparition frequencies for nodes were used as measures of species tree uncertainty.

*BEAST generates posterior samples by simultaneously estimating gene and species trees under a hierarchical coalescent model while allowing for independent evolutionary processes in each genomic region. BEAUTi version 1.7.5 [48] was used to create XML-formatted input files for *BEAST v1.7.5. Substitution models were chosen as previously described and were unlinked across genes with parameters estimated separately for each gene. As a needed prior in *BEAST, isolates were assigned to species groups under the Traits tab based on the three clades identified in the *endoPG* phylogeny. Evolutionary rates were estimated under a Yule process [68]. A Yule model was chosen as the species tree prior, which assumes a constant lineage birth rate for each branch in the tree. This tree prior is most suitable for trees describing the relationships between individuals from different species and is often thought of as describing the net rate of speciation [69]. Species tree estimations were carried out based on strict molecular clock assumption, following the methods of Heled and Drummond [48]. Data sets were run for 50 million generations in BEAST, sampling every 5,000 generations. Analyses were performed twice. Postburnin trees were combined with the program LogCombiner (BEAST v 1.6.0), and chains were assumed to converge when the average standard deviation of split frequencies was found to be < 0.011. The maximum clade credibility tree with posterior probability of each node was computed with the program TreeAnnotator (BEAST v 1.6.0). Log files were evaluated in TRACER version 1.5 [48]. The species tree was calculated using TreeAnnotator version 1.7.2 with a burn-in of 5000 trees. FigTree version 1.3.1 [70] was used to visualize the consensus tree node ages, branch lengths and posterior probabilities.

## Results

### Worldwide phylogenetic lineages

*Alternaria alternata* sampled from tangerine and tangerine hybrids on a worldwide scale (*n* = 142) revealed a total of 13 *endoPG* haplotypes (Additional file 1: Table S1, Figure 1A). Bayesian and maximum likelihood analyses of *endoPG* resulted in two phylogenetic lineages using a 95 posterior probability and 70 bootstrap value criterion (Figure 1B) [66]. These two lineages are subsequently referred to as ‘citrus 2’ and ‘citrus 3’. All other haplotypes (Hap8, Hap11, and Hap12) were placed into another group called citrus 1 based on previously published results that showed the existence of three lineages [35].

### Coalesence analyses

Thirty-five representative isolates were selected based on unique *endoPG* haplotype and geographical location to resolve the ancestral histories of the lineages using coalescent-based approaches. Neutrality and intra-locus recombination rates were estimated for all the genomic regions using D (Tajima’s and Fu and Li’s) statistics and Rmin. We were unable to reject the null hypothesis of neutrality for two loci (Table 1).

**Table 1:** Summary statistics of DNA polymorphisms in a worldwide sample of brown spot pathogen using four loci

**Locus (No. of isolates)**	***endoPG*** **(142)**^**W**^	***endoPG*** **(35)**	**OPA2-1 (35)**	**F3 (35)**	**OPA1-3 (35)**	**Combined (35)**
No. of sites (^A^)	427 (422)	427 (421)	538 (535)	222 (220)	503 (493)	1672 (1686)
No. of poly. sites (PI/PUI^B^)	21 (10/11)	24 (8/16)	20 (14/6)	15 (8/7)	75 (55/20)	135 (77/58)
No. of haplotypes^C^	13	13	7	7	12	24
Haplotype diversity	0.72	0.86	0.80	0.48	0.87	0.95
Nucleotide diversity	0.007	0.009	0.004	0.009	0.041	0.017
Tajima’s D	−0.78^NS^	−1.10^NS^	−1.70^NS^	−1.35^NS^	0.06^NS^	−0.62^NS^
Fu and Li’s D	−4.05^S^	−2.99^S^	−2.96^S^	−1.60^NS^	−0.16^NS^	−1.47^NS^
Rec events^R^	N/A	0	0	6	8	N/A

^A^Excluding indels.

^B^Number of polymorphic sites (informative/uninformative).

^C^Excludes outgroup isolate, BMP2031 (*A. tomato*).

^W^Includes all isolates in the worldwide dataset (ca.142).

^NS^Non-significant, ^S^Significant; *P* > 0.10.

^R^Number of recombination events estimated by Recom v. 5.8 [59].

**Figure 2:**
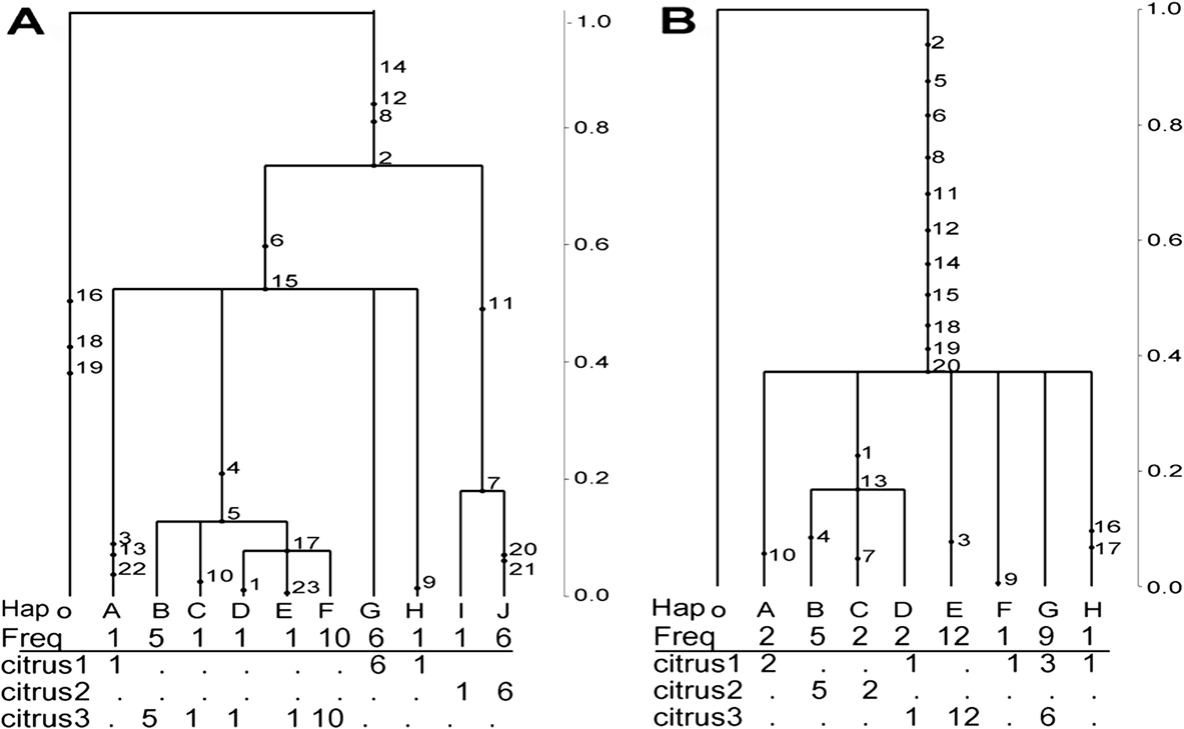
**Coalescent-based gene genealogies estimated assuming no recombination for loci (A) EndoPG and (B) OPA2-1, with no conflicting sites.** Each genealogy is scaled to TMRCA of 1 and numbers represent mutations. Each haplotype is labeled with a letter and the frequencies of each haplotype and relationship to each lineage is shown.

Coalescent-derived gene genealogies were estimated for the two non-recombining loci, *endoPG* and OPA2-1 (Figures 2A-B). In the *endoPG* genealogy, isolates of the citrus 1 lineage were positioned ancestral to all other haplotypes by several mutations (Figure 2A). In locus OPA2–1 signals of incomplete lineage sorting, where ancestral polymorphisms have not been sorted, were evident (Figure 2B). For example, some isolates from citrus 1 and citrus 3 were interspersed among identified clades, suggestive of non-reciprocal monophyly (Figure 2B). Further, little variation was revealed in the OPA2-1 genealogy, but eleven mutations, respectively, separated the *A. alternata* lineages from the *A. tomato* outgroup. Isolates from all lineages shared haplotypes, such that isolates from lineages citrus 1 and 3 formed a paraphyletic group. This was also observed in the OPA2-1, where haplotypes D and G included isolates from lineages citrus 1 and 3. Two clusters were formed, one including isolates from citrus 1 and 3, while the other included a polytomy of haplotypes from the citrus 1 and citrus 2 lineages (Figure 2B).

**Figure 3:**
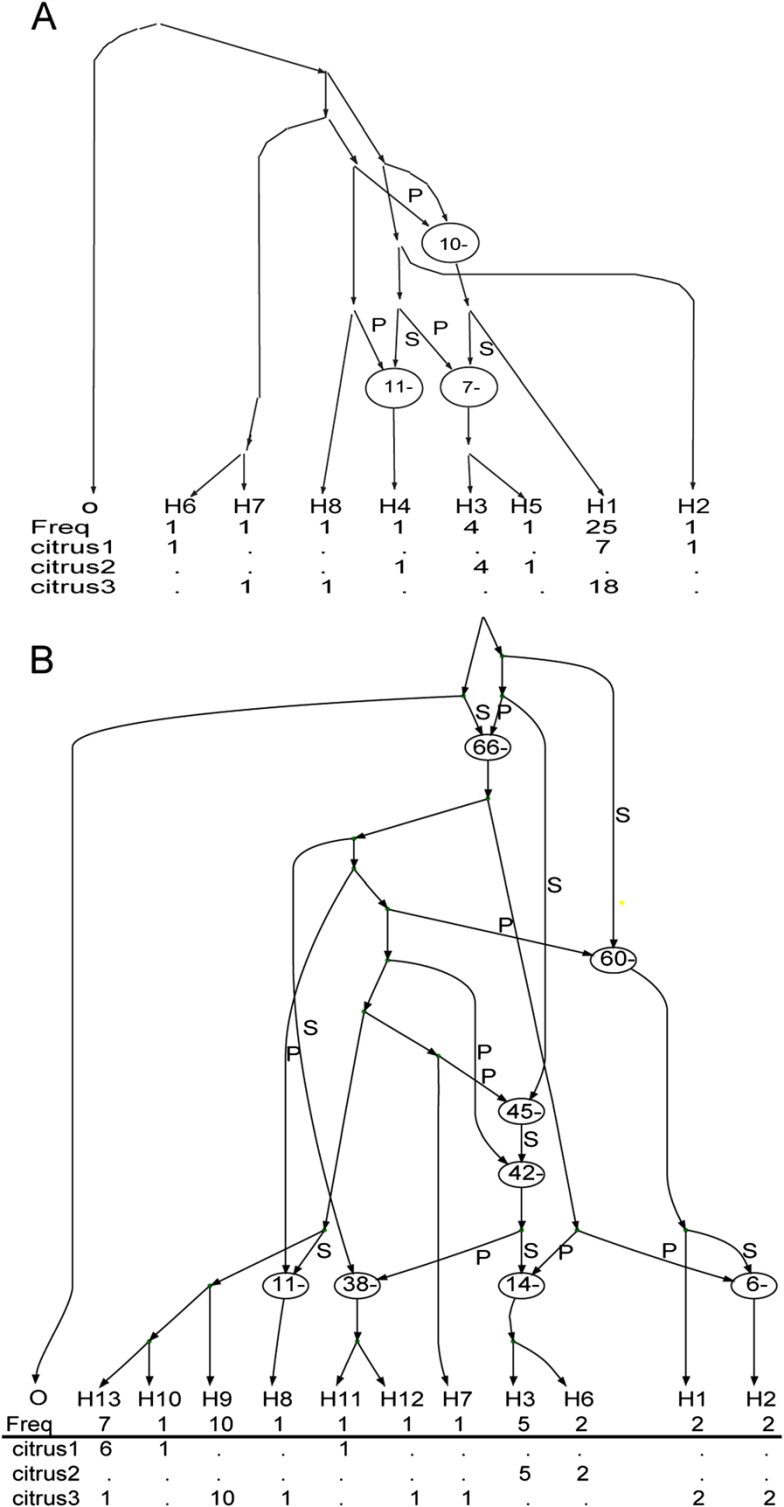
**Minimal ancestral recombination graphs for loci (A) Flank-F3 and (B) OPA1-3, rooted with*A. tomato*.** Recombination events are indicated by ovals. Recombinant sites consists of a prefix ‘P’ sequence that is concatenated with a suffix ‘S’ sequence. These designations appear on edges going into a recombination node. Numbers to the right/left of the paths are the number of mutations in that segment. The direction of the paths is from past to present.

Loci OPA1-3 and Flank-F3 had evolutionary histories of recombination. Three recombination events were observed in Flank-F3, which in all cases involved isolates from lineage citrus 1 and 2 (Figure 3A). OPA1–3 had the most complex evolutionary history involving eight recombination events (Figure 3B). These events included isolates from all three lineages suggesting possibly a sexual past as suggested by Berbee et al. [71].

### Phylogenetic analyses

**Figure 4:**
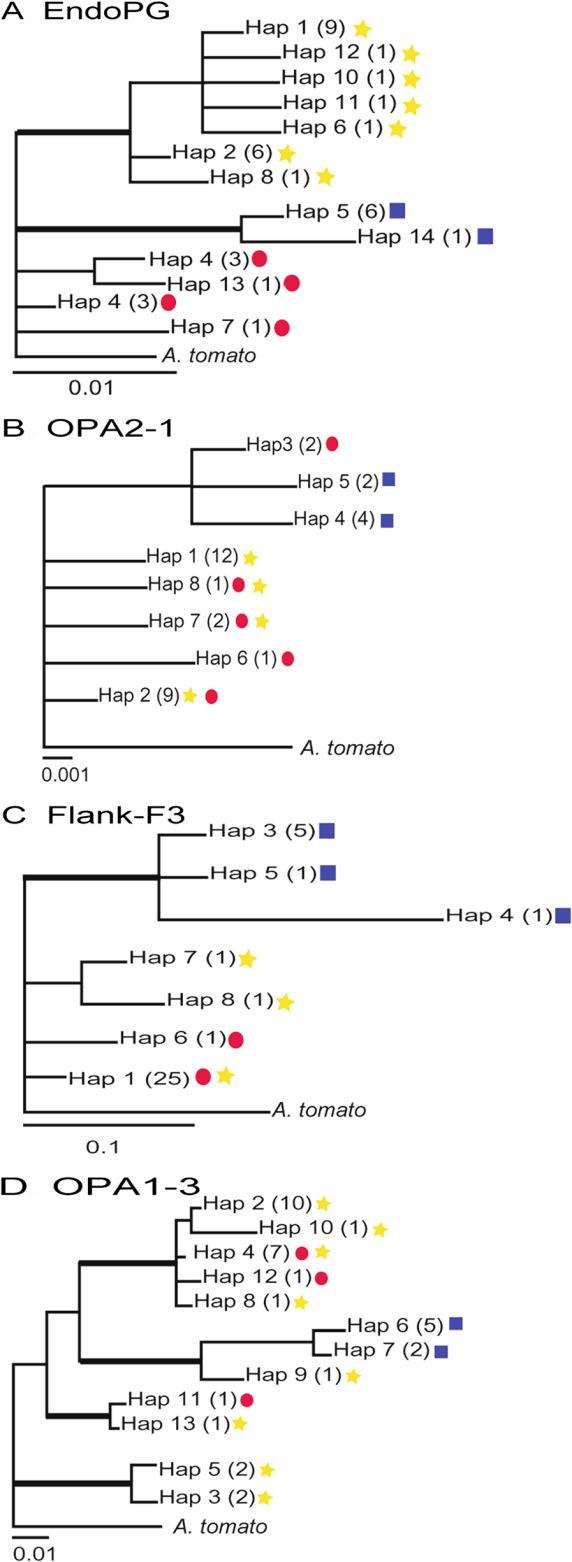
**Bayesian phylogenies estimated for each locus, (A)*endoPG*, (B) OPA2-1, (C) Flank-F3, and (D) OPA1-3.** Bold branches highlight nodes that were supported by 70% bootstrap values (maximum likelihood, 1000 replications) and 95 posterior probabilities. Numbers in parentheses represent the number of isolates of each haplotype. *EndoPG* haplotypes sampled from the citrus 1, citrus 2 and citrus 3 phylogenetic lineages are indicated by red circles, blue squares, and yellow stars, respectively.

Analyses were conducted for each locus independently for 35 isolates with representative *endoPG* haplotypes selected from the original worldwide sample. Analysis of *endoPG* yielded a phylogeny with similar topology to that estimated among the total worldwide sample, supporting three clades (Figure 4A). Eight haplotypes were identified in locus OPA2-1, however no monophyly was observed in the lineages (Figure 4B). A total of 7 haplotypes were identified locus Flank-3 (Figure 4C). Only one well-supported clade was found, including all isolates from citrus 2. Locus OPA1-3, with 13 haplotypes, had the most phylogenetic resolution, resulting in six well-supported clades (Figure 4D). Among 503 sites, 75 sites (14.7%) were polymorphic. Of these, 55 (10.9%) were parsimony informative (Table 1). Of these clades, one corresponded to a terminal clade including all citrus 2 haplotypes, clearly separated from but sister to one citrus 1 haplotype, and polyphyletic relationships were observed in lineages 1 and 3 (Figures 4A-D).

### Divergence time estimation for the endoPG

A total of six synonymous nucleotide changes were observed when comparing haplotypes of the citrus lineages 1 and 3 against haplotypes from citrus 2. Though only a portion of the CDS region was analyzed (427 out of 1137 bp), using the published rates for neutral gene substitution rate of 0.9 × 10^−9^ and 16.7 × 10^−9^[72] resulted in a divergence date of at least 5,400 thousand years before present.

### Estimation of species trees from phylogenetically incongruent gene genealogies

In the phylogenies from the concatenated and individual datasets, lineages citrus 1 and 3 were polyphyletic. Support for the citrus 2 lineage was found in most gene trees when GCPSR criteria was applied (Figure 4A-D). The citrus 1 and 3 lineages were only well-supported in the *endoPG* phylogeny and thus constituted a single, polyphyletic species when the other loci were considered.

**Figure 5:**
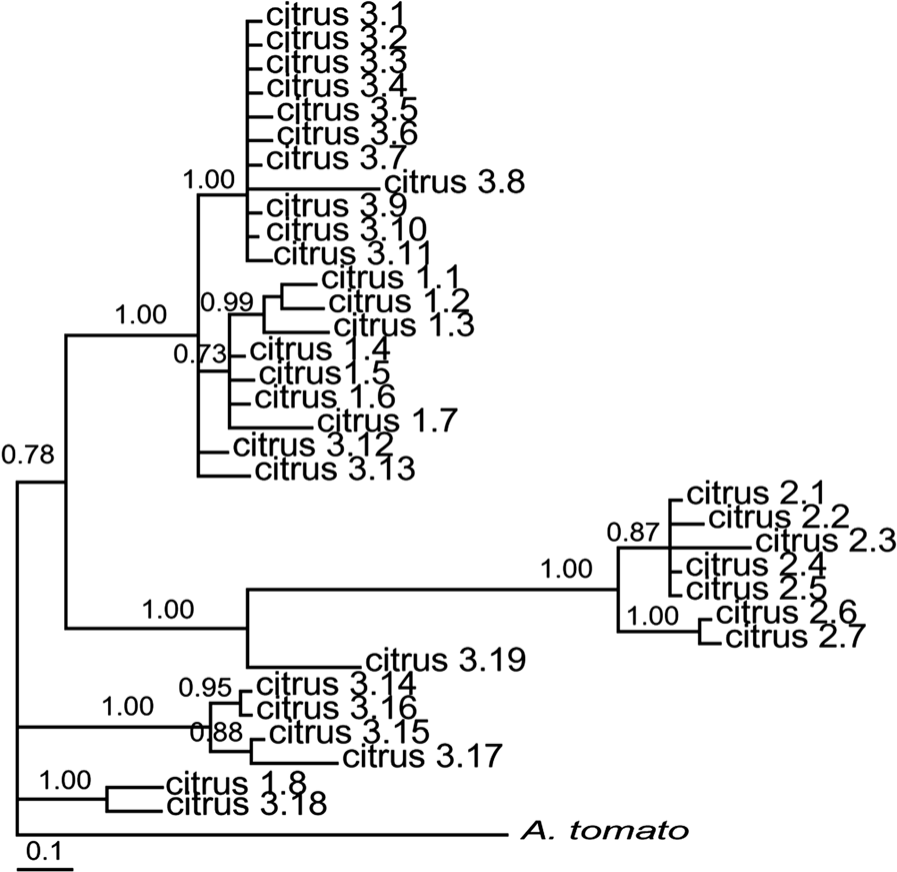
**Bayesian phylogeny derived from the concatenated dataset with a total of 1672 nucleotides from four loci including the*endo-polygalacturonase*gene, SCAR markers OPA2-1 and OPA1-3, and one microsatellite flanking region AA-Flank-3.** Node support in posterior probability is indicated for each node.

**Figure 6:**
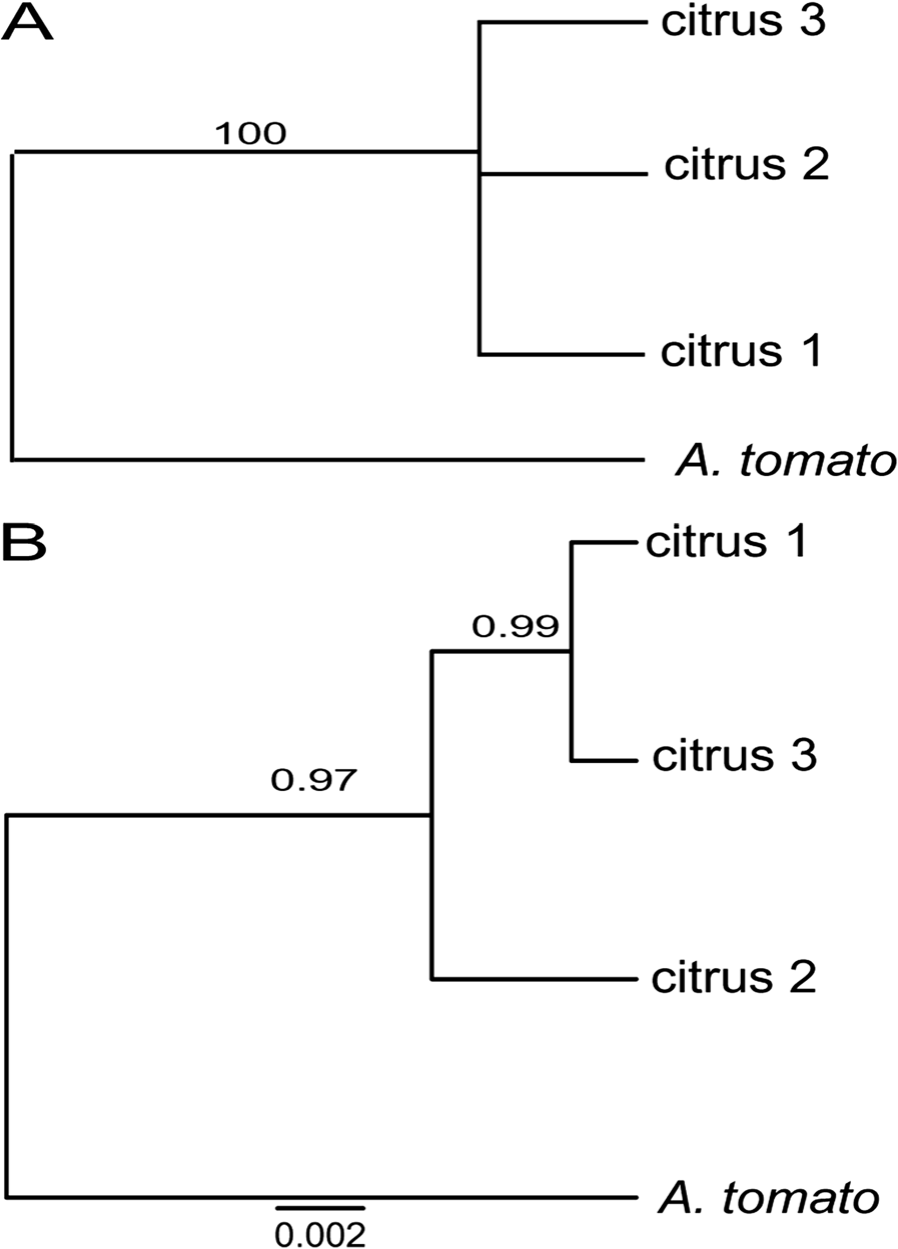
Species tree estimations among a worldwide sample of citrus brown spot pathogen species groups using (A) the “minimize deep coalescent’ (MDC) approach, and a hierarchical Bayesian model as implemented in (B) *BEAST. Numbers above branches indicate node support as posterior probabilities.

Congruence of tree topology was tested and statistically significant incongruence was detected for all pairwise comparisons of all loci with OPA1-3 (*P* = 0.00) using Shimodaira-Hasegawa tests (Table 2). All other pairwise comparisons were non-significant.The phylogeny generated from the concatenated dataset yielded four well-supported clades with topology similar to the endoPG phylogeny (Figure 5). The citrus 1 and 3 lineages were again polyphyletic, with individuals from each falling into three clades. However, lineage citrus 2 separated into one monophyletic well-supported (1.00 posterior probability) lineage.Little support for multiple species was obtained using the MDC approach. In the MDC-estimated species tree, all lineages clustered into one clade with high bi-partition frequency (over 98%) (Figure 6A). In contrast, *BEAST analyses revealed two well-supported species, citrus 2 and a second species comprising lineages citrus 1 and 3 (Figure 6B).

**Table 2:** Pairwise Shimodaira-Hasegawa tests of topological congruence among phylogenies

	**OPA2-1**	**OPA1-3**	**Flank-F3**
*endoPG*	0.059 ^a^	0.000	0.063
OPA2-1		0.000	0.128
OPA1-3			0.000

^a^probability of different topologies among 2000 bootstrapped datasets generated using RELL sampling.

## Discussion

Coalescent-based approaches and ancestral recombination graphs were used to elucidate the evolutionary history of the citrus brown spot pathogen on a worldwide scale and quantitatively estimate the number of species causing this disease. The evolutionary history of these lineages showed patterns of incomplete lineage sorting and recombination as has been observed among other closely-related taxa [10,14] and was hinted at in previous phylogenetic studies of *Alternaria*[42]. Lineage sorting, recombination, and horizontal transfer [17] make phylogenetic analyses and species delimitation among small-spored *Alternaria* challenging [28,36,40]. In contrast to previous studies that described ten morphospecies causing Alternaria brown spot of citrus [34] we identified one or two species among three phylogenetic lineages using the “minimize deep coalescence” (MDC) and hierarchical Bayesian model approaches, respectively. Both methods failed to differentiate the citrus 1 and 3 lineages but the hierarchical Bayesian model method differentiated the citrus 2 lineage from citrus 1 and 3. Species boundaries between the morphospecies *A. citriarbusti* (citrus 1) and *A. dumosa*, *A. turkisafria*, *A. perangusta* and *A. interrupta* (citrus 3) were poorly supported by both methods. However, the GCPSR and *BEAST analyses separated the morphospecies *A. tangelonis* and *A. colombiana* (citrus 2) from citrus 1 and 3. The hypothesis that all host-specific toxin producing, small-spored *Alternaria* taxa infecting citrus as well as other hosts such as strawberry, Japanese pear, apple, and tomato represent sub-specific variants or “pathotypes” of *A. alternata* is not new [73]. One of our analyses supported this hypothesis and all other analyses conclusively demonstrated that the number of taxa causing brown spot disease of citrus has been over-estimated using morphological criteria. Further study is required to determine if the citrus 2 lineage represents a distinct species, and if so, what these two species should be named.

### Evolution of the citrus pathogens

*Alternaria alternata* is an interesting model with which to study speciation in putatively asexual taxa. Asexual taxa do not easily fit within species concepts developed for sexual taxa [2,4,8,74,75]). In addition to being a well-recognized saprotroph, *A. alternata* infects a wide range of hosts, including citrus, pear, strawberry, and apple [73,76,77]. Pathogenic forms of *A. alternata* are thought to have radiated from a recent common saprophytic ancestor through the horizontal acquisition of pathogenicity factors [73,78]. Isolates sampled for this study are considered representative of the “tangerine pathotype” [38,79,80] and these fungi produce host-specific ACT-toxins that are required for pathogenicity [74,81]. ACT-toxins are structurally similar to the host-specific toxins produced by strawberry pathotype (AFT-toxins) and Japanese pear pathotype isolates (AKT-toxins) [81] and the genes controlling the biosynthesis of these toxins are homologous [81,82] Connecting the three citrus lineages to the lineages of strawberry and Japanese pear pathotype isolates in future studies will allow polarization of the phylogeny of host-specific toxin-producing *Alternaria* and facilitate studies of toxin gene evolution and the evolution of pathogenicity. Assuming that the mutation rate for the *endoPG* gene is similar to other protein coding genes at approximately 0.9 × 10^−9^ to 16.7 × 10^−9^ mutations per site per year [71,72,83] the estimated time of divergence of the citrus 2 lineage and the citrus 1 and 3 lineages is at least 5, 400 years before present, and probably occurred much earlier. This date suggests that divergence of citrus 2 occurred long before the movement of citrus, and presumably its pathogens, from its putative center of origin in southeastern Asia less than 600 years ago [84]. To date, only citrus 2 isolates have been isolated from North and South America, whereas the other lineages are found on several Old and New World continents. This suggests that independent introductions of each lineage may have occurred in different locations, presumably with the host.

Using GCPSR criteria, many cryptic species have been identified, including several plant pathogenic fungi [85-87], human-infecting fungi [88-90], and an insect pathogen [11]. Latin names have been assigned to some of these cryptic species [91-93]. As far as we are aware, this is the first report that compares the GCPSR method to other newly developed tree species estimation methods. Our results suggest that in the presence of divergent evolutionary histories, GCPSR will likely overestimate the number of species. Although no reports could be found comparing the GCPSR method with others, several studies have compared species tree estimates generated with *BEAST and/or MDC to concatenated results. It is now well-documented that concatenated data can produce well-supported phylogenies that are inconsistent with the true species tree [13,94-96]. Furthermore, Belfiore et al. [97] developed species trees using concatenation and BEST (Bayesian Estimation of Species Tree) for pocket gophers of the genus *Thomomys* and found that the concatenated tree estimated from seven loci was over-resolved whereas fewer species were supported in the phylogeny estimated using BEST [97]. This result is similar to what we observed in this study with *A. alternata*. Further, Eckert and Carstens [11] tested the accuracy of concatenation and MDC generated species trees using simulated datasets in the presence of gene flow. As the level of gene flow increased the probability of identifying the true species using concatenation dropped to zero, although this value only decreased to 74% using MDC [11]. We found similar results, with the *BEAST and concatenated trees having similar topologies, but with node support varying widely. Our results suggest that species tree estimation methods that account for gene tree uncertainty among loci with diverged histories, with signals of lineage sorting and recombination may result in fewer well-supported species than concatenation, especially among closely related fungal taxa.

Two of the loci we employed in this study (Flank-F3 and OPA1-3) revealed strong evidence for a history of recombination. Asexual *Alternaria* species are thought to be derived from sexual ancestors [71], as has been suggested for other asexual ascomycetes [98], and one species *A. infectoria* has been connected to a *Lewia* teleomorph [34]. Although time scales for our ancestral recombination graphs (ARG) are not possible, the ARG for Flank-F3 showed three possible recombination events where citrus 2 haplotypes are possibly derived from citrus lineages 1 and 3. It is difficult to date these putative recombination events and to know if these events are the result of historic or current sexual or asexual recombination. Signatures of recombination were found in the citrus 1 lineage in an *Alternaria* brown spot fungus population in Florida [29] but the mechanism of this recombination is not known. *Alternaria* may be able to recombine through parasexual and/or sexual means [29], which concurs with the findings that mating type genes in *A. alternata* are expressed [5] and under strong purifying selection [99]. Further studies will be needed to determine if *Alternaria alternata* sensu lato is capable of forming a sexual stage than has heretofore been overlooked. If so, results from our ARG may represent contemporary rather than historical recombination, although dating recombination events in the ARG is not possible.

## Conclusions

Species delimitation is important for the study of the evolution of pathogenicity and the emergence of infectious diseases. Further, the delimitation of species also plays a critical role in global biosecurity by providing guidelines for restrictions on the movement of plant pathogens among countries which has national and international significance [100,101]. The threat of movement of introduced pathogens around the world has resulted in the quarantine of many crops or the rejection of exported crops. Incorrectly naming a new species or wrongly identifying a species can result in significant economic losses [102]. In 2001–2002, shipments of Li Ya pear imported from China were rejected because of signs of *Alternaria* spp. infection, which also occurred in Australia, New Zealand, and United Kingdom. Two related species, *A. alternata* and *A. gaisen*, were included in a pest risk assessment and further research showed that isolates sampled from Li Ya pear were morphologically distinct and given a new name, *A. yaliinficiens*[100]. Our results indicate that citrus brown spot is caused by a maximum of two species of *Alternaria*, and that taxonomic revision of *Alternaria* infecting citrus, based on congruent morphological and genetic analyses, is needed. One of these species (encompassing lineages citrus 1 and citrus 3) is found worldwide but the second (lineage citrus 2) species has only been found in the Americas. It is not yet known if phenotypic differences in aggressiveness, host range, or growth rates exist between these species, but if so, limiting the movement of the citrus 2 species into new countries might be warranted. This study highlights the need for the use of these new methods to accurately identify closely related, morphologically indistinguishable species that are important in agriculture and potentially of regulatory interest.

### Availability of supporting data

The data sets supporting the results of this article are available in GenBank under accession numbers KF699389-KF699527.

## Competing interests

The authors declare that they have no competing interests.

## Authors’ contributions

JES and TLP conceived of the study. JES carried out molecular genetic studies and bioinformatics, and analyses. JES, TLP, LPT, CML, and BMP wrote and edited the manuscript. All authors read and approved the final manuscript.

## Supplementary Material

Additional file 1: Table S1 Isolates used in the study [103,105]. Click here for file

## References

[B1] Perkins DD Bennett JW, Lasure LL In Praise of Diversity More Gene Manipulations in Fungi 1991 New York: Academic Press 3 26

[B2] Harrington TC Rizzo DM Worrall JJ Defining species in the fungi Structure and Dynamics of Fungal Populations 1999 Dordrecht, The Netherlands: Kluwer Press 43 71

[B3] Maynard Smith J Szathmáry E The Major Transitions in Evolution 1995 London: Oxford University Press

[B4] Kohn LM Mechanisms of fungal speciation Annu Rev Phytopathol 2005 43 279 308 10.1146/annurev.phyto.43.040204.135958 16078886

[B5] Taylor JW Jacobson DJ Kroken S Kasuga T Geiser DM Hibbett DS Fisher MC Phylogenetic species recognition and species concepts in fungi Fungal Genet Biol 2000 31 21 32 10.1006/fgbi.2000.1228 11118132

[B6] Hibbett DS Taylor JW Fungal systematics: is a new age of enlightenment at hand? Nat Rev Microbiol 2013 11 129 133 10.1038/nrmicro2963 23288349

[B7] Hawsworth DL Misra JK, Tewari JP, Deshmukh SK Integrating Morphological and Molecular Data in Fungal Systematics Systematics and Evolution of Fungi 2012 New York, NY: CRC Press 1 14

[B8] Kohn LM The clonal dynamic in wild and agricultural plant populations Can J Bot 1995 73 S1231 S1240 10.1139/b95-383

[B9] Taylor JW Jacobson DJ Fisher MC The evolution of asexual fungi: reproduction, speciation, and classification Annu Rev Phytopathol 1999 37 197 246 10.1146/annurev.phyto.37.1.197 11701822

[B10] Carstens BC Knowles LL Estimating species phylogeny from gene-tree probabilities despite incomplete lineage sorting: An example from Melanoplus grasshoppers Syst Biol 2007 56 400 411 10.1080/10635150701405560 17520504

[B11] Eckert AJ Carstens BC Does gene flow destroy phylogenetic signal? The performance of three methods for estimating species phylogeneies in the presence of gene flow Mol Phylogenet Evol 2008 49 832 842 10.1016/j.ympev.2008.09.008 18845264

[B12] Kubatko LS Carstens BC Knowles LL STEM: species tree estimation using maximum likelihood for gene trees under coalescence Bioinformatics 2009 25 9 973 10.1093/bioinformatics/btp079 19211573

[B13] Kubatko LS Degnan JH Inconsistency of phylogenetic estimates from concatenated data under coalescence Syst Biol 2007 56 17 24 10.1080/10635150601146041 17366134

[B14] Maddison WP Gene trees in species trees Syst Biol 1997 46 523 536 10.1093/sysbio/46.3.523

[B15] Hartl DL Clark AG Principles of Population Genetics 2007 4 Sinauer Associates, Inc: Sunderland, MA

[B16] Degnan JH Salter LM Gene tree distributions under the coalescent process Evolution 2005 9 24 37 15792224

[B17] Maddison WP Knowles LL Inferring phylogengy despite incomplete lineage sorting Syst Biol 2006 55 410 431 10.1080/10635150500354928 16507521

[B18] Lui L Pearl DK Species trees from gene trees: Reconstructing Bayesian posterior distributions of a species phylogeny using estimated gene tree distributions Syst Biol 2007 56 504 514 10.1080/10635150701429982 17562474

[B19] Niemiller ML Fitzpatrick BM Miller BT Recent divergence with gene flow in Tennessee cave salamanders (Plethodontidae: Gyrinophilus) inferred from gene genealogies Mol Ecol 2008 17 2258 2275 10.1111/j.1365-294X.2008.03750.x 18410292

[B20] Brumfield RT Liu L Lum DE Edwards SV Comparison of species tree methods for reconstructing the phylogeny of bearded manikins (Aves: Pipridae: *Manacus*) from multilocus sequence data Syst Biol 2008 57 9 731 10.1080/10635150802422290 18853359

[B21] Cranston KA Hurwitz B Ware D Stein L Wing RA Species trees from highly incongruent gene trees in rice Syst Biol 2009 58 489 500 10.1093/sysbio/syp054 20525603

[B22] Henk DA Eagle CE Brown K VanDenBerg MA Dyer PS Peterson SW Fisher MC Speciation despite globally overlapping distributions in *Penicillium chyrysogenum*: the population genetics of Alexander Fleming’s lucky fungus Mol Ecol 2011 20 4288 4301 10.1111/j.1365-294X.2011.05244.x 21951491

[B23] Pollard DA Iyer VN Moses AM Eisen MB Widespread discordance of gene trees with species tree in Drosophila: evidence for incomplete lineage sorting PLoS Genet 2006 2 e173 10.1371/journal.pgen.0020173 17132051 PMC1626107

[B24] Browne AGP Fisher MC Henk DA Species-specific PCR to describe local-scale distribution of four cryptic species in the *Penicillium chrysogenum* complex Fungal Ecol 2013 6 419 429 10.1016/j.funeco.2013.04.003 24179477 PMC3809933

[B25] Pamilo P Nei M Relationships between gene trees and species trees Mol Biol Evol 1988 5 568 583 3193878 10.1093/oxfordjournals.molbev.a040517

[B26] Leache AD Species tree discordance traces to phylogeographic clade boundaries in North American fence lizards (*Sceloporus*) Syst Biol 2008 56 547 559 10.1093/sysbio/syp057 20525608

[B27] Linnen CR Species tree estimation for complex divergence histories: a case study in Neodiprion sawflies. In Estimating Species Trees. Edited by Knowles LL, Kubatko L 2010 New York: Wiley-Blackwell

[B28] Andrew M Peever TL Pryor BM An expanded multilocus phylogeny does not resolve morphological species within the small-spored *Alternaria* species complex Mycologia 2009 101 95 109 10.3852/08-135 19271672

[B29] Stewart JE Thomas KA Lawrence CB Dang H Pryor BM Peever TL **Signatures of recombination in clonal lineages of the citrus brown spot pathogen,**** *Alternaria alternata* ****sensu lato.** Phytopathology 2013 103 741 749 10.1094/PHYTO-08-12-0211-R 23441968

[B30] Pierce NB Black rot of oranges Bot Gazelle 1902 33 234 23 10.1086/328217

[B31] Neergaard P Danish Species of Alternaria and Stemphylium 1945 London: Oxford University Press 560

[B32] Ellis MB Dematiaceous Hyphomycetes Kew: Commonwealth Mycological Institute 19

[B33] Kusaba M Tsuge T Nuclear ribosomal DNA variation and pathogenic specialization in *Alternaria* fungi known to produce host-specific toxins Appl Environ Microbiol 1994 60 3055 3062 16349367 10.1128/aem.60.9.3055-3062.1994 PMC201771

[B34] Simmons EG Alternaria themes and variations (226–235): Classification of citrus pathogens Mycotaxon 1999 70 263 323

[B35] Peever TL Ibañez A Akimitsu K Timmer LW Worldwide Phylogeography of the citrus brown spot pathogen, *Alternaria alternata* Phytopathology 2002 92 794 802 10.1094/PHYTO.2002.92.7.794 18943277

[B36] Peever TL Su G Carpenter-Boggs L Timmer LW Molecular systematics of citrus-associated *Alternaria* spp Mycologia 2004 96 119 134 10.2307/3761993 21148834

[B37] Kingman JFC The coalescent Stoch Process Appl 1982 13 235 248 10.1016/0304-4149(82)90011-4

[B38] Peever TL Canihos Y Olsen L Ibanez A Liu Y–C Timmer LW Population genetic structure and host specificity of Alternaria spp. causing brown spot on Minneola tangelo and rough lemon in Florida Phytopathology 1999 92 794 802 18944727 10.1094/PHYTO.1999.89.10.851

[B39] Isshiki A Akimitsu K Yamamoto M Yamamoto H Endopolygalacturonase is essential for citrus black rot caused by *Alternaria citri* but not brown spot caused by *Alternaria alternata* Mol Plant-Microbe Interact 2001 14 749 757 10.1094/MPMI.2001.14.6.749 11386370

[B40] Peever TL Carpenter-Boggs L Timmer LW Carris LM Bhatia A Citrus black rot is caused by phylogenetically distinct lineages of *Alternaria alternata* Phytopathology 2005 95 512 518 10.1094/PHYTO-95-0512 18943316

[B41] Su G Peever TL Timmer LW Molecular systematics of citrus-associated *Alternaria sp* Phytopathology 2001 91 S190

[B42] Andrew M Molecular systematics of small-spored Alternaria species 2006 MS Thesis: Washington State University

[B43] Tran-Dinh N Hocking A Isolation and characterization of polymorphic microsatellite markers for *Alternaria alternata* Mol Ecol Resour 2006 6 405 407

[B44] Rozen S Skaletsky H Krawetz S Misener S Primer3 on the WWW for general users and for biologist programmers In bioinformatics Methods and Protocols 2000 365 386 10.1385/1-59259-192-2:365 10547847

[B45] Guindon S Gascuel O A simple, fast, and accurate algorithm to estimate large phylogenies by maximum likelihood Syst Biol 2003 52 696 707 10.1080/10635150390235520 14530136

[B46] Huelsenbeck JP Ronquist F MrBayes: Bayesian inference of phylogenetic trees Bioinformatics 2001 17 754 755 10.1093/bioinformatics/17.8.754 11524383

[B47] Minin V Abdo Z Joyce P Sullivan J Performance-based selection of likelihood models for phylogeny estimation Syst Biol 2003 52 674 683 10.1080/10635150390235494 14530134

[B48] Heled J Drummond AJ Bayesian inference of species trees from multilocus data Mol Biol Evol 2010 2010 27 570 580 19906793 10.1093/molbev/msp274 PMC2822290

[B49] Shimodaira H Hasegawa M Multiple comparisons of loglikelihoods with applications to phylogenetic inference Mol Biol Evol 1999 16 1114 1116 10.1093/oxfordjournals.molbev.a026201

[B50] Swofford DL PAUP*. Phylogenetic Analysis Using Parsimony (*and Other Methods). Version 4 2003 Sunderland, Massachusetts: Sinauer Associates

[B51] Hall TA BioEdit: a user-friendly biological sequence alignment editor and analysis Nucleic Acids Symp Ser 1999 41 95 98

[B52] Librado P Rozas J DnaSP v5: A software for comprehensive analysis of DNA polymorphism Bioinformatics 2009 25 1451 1452 10.1093/bioinformatics/btp187 19346325

[B53] Rozas J Sanchez-Del J Barrio JC Messeguer X Rozas R DnaSP, DNA polymorphism analyses by the coalescent and other methods Bioinformatics 2003 19 2496 2497 10.1093/bioinformatics/btg359 14668244

[B54] Hudson RR Kaplan NL Statistical properties of the number of recombination events in the history of a sample of DNA sequences Genetics 1985 111 147 164 4029609 10.1093/genetics/111.1.147 PMC1202594

[B55] Jakobsen IB Easteal S A program for calculating and displaying compatibility matrices as an aid in determining reticulate evolution in molecular sequences Comput Appl Biosci 1996 12 291 295 8902355 10.1093/bioinformatics/12.4.291

[B56] Price EW Carbone I SNAP: workbench management tool for evolutionary population genetics analysis Bioinformatics 2005 21 402 404 10.1093/bioinformatics/bti003 15353448

[B57] Aylor DL Price EW Carbone I SNAP: combine and map modules for multilocus population genetics analysis Bioinformatics 2006 22 1399 1401 10.1093/bioinformatics/btl136 16601003

[B58] Hey J Wakely J A coalescent estimator of the population recombination rate Genetics 1997 145 833 846 9055092 10.1093/genetics/145.3.833 PMC1207867

[B59] Griffiths RC Marjoram P Ancestral inference from samples of DNA sequences with recombination J Comput Biol 1996 3 479 502 10.1089/cmb.1996.3.479 9018600

[B60] Lawrence DP Gannibal PB Peever TL Pryor BM The sections of Alternaria: formalizing species-group concepts Mycologia 2013 105 530 546 10.3852/12-249 23687125

[B61] Bahlo M Griffiths RC Inference from gene trees in a subdivided population Theor Popul Biol 2000 57 79 95 10.1006/tpbi.1999.1447 10792974

[B62] Griffiths RC Tavaré S Ancestral inference in population genetics Stat Sci 1994 9 307 319 10.1214/ss/1177010378

[B63] Lyngso RB Song YS Hein J Minimum Recombination Histories by Branch and Bound Algorithms in Bioinformatics, Proceedings 2005 Berlin: Springer-Verlag Berlin 239 250

[B64] Oliver JC AUGIST: inferring species trees while accommodating gene tree uncertainty Bioinformatics 2008 24 2932 2933 10.1093/bioinformatics/btn556 18953045 PMC2639298

[B65] Avise JC Ball RM Jr Principles of genealogical concordance in species concepts and biological taxonomy Oxf Surv Evol Biol 1990 7 45 67

[B66] Alfaro ME Zoller S Lutzoni F Bayes or bootstrap? A simulation studying comparing the performance of Bayseian markov chain Monte Carlo sampling and bootstrapping in assessing phylogenetic confidence Mol Biol Evol 2003 20 255 266 10.1093/molbev/msg028 12598693

[B67] Maddison WP Maddison DR Mesquite: a modular system for evolutionary analysis. Version 2.73 2010 http://mesquiteproject.org

[B68] Yule GU A mathematical theory of evolution, based on the conclusions of Dr. J.C. Willis, F.R.S Philos Trans R Soc Lond Biol 1925 213 21 87 10.1098/rstb.1925.0002

[B69] Rannala B Yang Z Probability distribution of molecular evolutionary tree: a new methods for phylogenetic inference J Mol Evol 1996 43 304 311 10.1007/BF02338839 8703097

[B70] Rambaut A FigTree, a graphical viewer of phylogenetic trees 2008 http://tree.bio.ed.ac.uk/software/figtree

[B71] Berbee ML Payne BP Zhang G Roberts RG Turgeon BG Shared ITS DNA substitutions in isolates of opposite mating type reveal a recombining history for three presumed asexual species in the filamentous ascomycete genus *Alternaria* Mycol Res 2003 107 169 182 10.1017/S0953756203007263 12747328

[B72] Kasuga T White TJ Taylor JW Estimation of nucleotide substitution rates in Eurotiomycete fungi Mol Biol Evol 2002 19 2318 2324 10.1093/oxfordjournals.molbev.a004056 12446823

[B73] Nishimura S Kohmoto K Host-specific toxins and chemical structures from *Alternaria* species Annu Rev Phytopathol 1983 21 87 116 10.1146/annurev.py.21.090183.000511 25946338

[B74] Chaverri P Castlebury LA Samuels GJ Geiser DM Multilocus phogenetics structure within the *Trichoderma harzianum*/*Hypocrea lixii* complex Mol Phylogenet Evol 2003 27 302 313 10.1016/S1055-7903(02)00400-1 12695093

[B75] Miller AN Huhndorf SM Using phylogenetic species recognition to delimit species boundaries and species relationships within *Lasiosphaeria* Mycologia 2004 96 302 313 21148930

[B76] Kohmoto K Otani H Tsuge T Kohmoto K, Singh US, Singh RP** *Alternaria alternata* ****Pathogens.** Pathogenesis and Host Specificity in Plant Diss: Histopathological, Biochemical, Genetic and Molecular Bases. Vol. II, Eukaryotes 1995 Oxford: Pergamon Press 51 63

[B77] Timmer LW Peever TL Solel Z Akimitsu K *Alternaria* diseases of citrus – Novel pathosystems Phytopathol Mediterrian 2003 42 3 16

[B78] Nishimura S Kohmoto K Otani H Ramachandran P Tamura F Asada Y, Bushnell WR, Ouchi S, Vance CP Pathological and epidemiological aspects of *Alternaria alternata* infection depending on a host-specific toxin Plant infection: the Physiological and Biochemical Basis 1982 Tokyo; Berlin: Japan Scientific Societies Press; Springer-Verlag 199 21

[B79] Akamatsu H Taga M Kodama M Johnson R Otani H Kohmoto K Molecular karyotypes for Alternaria plant pathogens known to produce host-specific toxin Curr Genet 1999 35 647 656 10.1007/s002940050464 10467010

[B80] Masunaka A Ohtani K Peever TL Timmer LW Tsuge T Yamamoto M Akimitsu K An isolate of Alternaria alternata that is pathogenic to both tanginess and rough lemon produces two host-selective toxins ACT and ACR toxins Phytopathology 2005 95 241 247 10.1094/PHYTO-95-0241 18943116

[B81] Masunaka A Tanaka A Tsuge T Peever TL Timmer LW Yamamoto M Yamamoto H Akimitsu K Distribution and characterization of AKT homologs in the tangerine pathotype of *Alternaria alternata* Phytopathology 2000 90 762 768 10.1094/PHYTO.2000.90.7.762 18944496

[B82] Tsuge T Harimoto Y Akimitsu K Ohtani K Kodama M Akagi Y Mayumi E Yamamoto M Otani H **Host-selective toxins produced by the plant pathogenic fungus**** *Alternaria alternata* ****.** FEMS Microbiol Rev 2013 37 44 66 10.1111/j.1574-6976.2012.00350.x 22846083

[B83] Li W-H Tanimura M Sharp PM An evaluation of the molecular clock hypothesis using mammalian DNA sequences J Mol Evol 1987 25 330 342 10.1007/BF02603118 3118047

[B84] Scora RW On the history and origin of citrus Torrey Bot Club 1975 102 369 375 10.2307/2484763

[B85] O’Donnell K Molecular phylogeny of the *Nectria haematococca*—*Fusarium solani* species complex Mycologia 2000 92 919 938 10.2307/3761588

[B86] O’Donnell K Kistler HC Tacke BK Casper HH Gene genealogies reveal global phylogeographic structure and reproductive isolation among lineages of *Fusarium graminearum*, the fungus causing wheat scab Proc Natl Acad Sci U S A 2000 97 7905 7910 10.1073/pnas.130193297 10869425 PMC16643

[B87] Steenkamp ET Wingfield BD Desjardins AE Cryptic speciation in *Fusarium subglutinans* Mycologia 2002 94 1032 1043 10.2307/3761868 21156574

[B88] Koufopanou V Burt A Taylor JW **Concordance of gene genealogies reveals reproductive isolation in the pathogenic fungus**** *Coccidioides immitis* ****.** Proc Natl Acad Sci U S A 1997 94 5478 5482 10.1073/pnas.94.10.5478 9144263 PMC24704

[B89] Kasuga T Taylor JW White TJ Phylogenetic relationships of varieties and geographical groups of the human pathogenic fungus, *Histoplasma capsulatum* Darling J Clin Microbiol 1999 37 653 663 9986828 10.1128/jcm.37.3.653-663.1999 PMC84508

[B90] Cruse M Telerant R Gallagher T Lee T Taylor JW Cryptic species in *Stachybotrys chartarum* Mycologia 2002 94 814 822 10.2307/3761696 21156555

[B91] Fisher MC Koenig G White TJ Taylor JW **Molecular and phenotypic description of**** *Coccidioides posadasii* ****sp. nov., previously recognized as the non-California population of**** *Coccidioides immitis* ****.** Mycologia 2002 94 73 84 10.2307/3761847 21156479

[B92] Couch BC Kohn LM **A multilocus gene genealogy concordant with host preference indicates segregation of a new species,**** *Magnaporthe oryzae* ****,**** *from* **** *M. grisea.* **Mycologia 2002 94 683 693 10.2307/3761719 21156541

[B93] O’Donnell K Ward TJ Geiser DM Kistler HC Aoki T Genealogical concordance between the mating type locus and seven other nuclear genes supports formal recognition of nine phylogenetically distinct species within the Fusarium graminearum clade Fungal Genet Biol 2004 41 600 623 10.1016/j.fgb.2004.03.003 15121083

[B94] Degnan JH Rosenberg NA Discordance of species trees with their most likely gene trees PLoS Genet 2006 2 762 768 10.1371/journal.pgen.0020068 PMC1464820 16733550

[B95] Rosenberg NA Tao R Discordance of species trees with their most likely gene trees: the case of five taxa Syst Biol 2008 57 131 140 10.1080/10635150801905535 18300026

[B96] Sen D Brown CJ Top EM Sullivan J Inferring the evolutionary history of IncP-1 plasmids despite incongruence among backbone gene trees Mol Biol Evol 2013 30 154 166 10.1093/molbev/mss210 22936717 PMC3525142

[B97] Belfiore NM Liu L Moritz C Multilocus phylogenetics of a rapid radiation in the genus *Thomomys* (Rodentia: Geomyidae) Syst Biol 2008 57 294 10.1080/10635150802044011 18432550

[B98] Yun SH Berbee ML Yoder OC Turgeon BG Evolution of the fungal self-fertile reproductive life style from self-sterile ancestors Proc Natl Acad Sci U S A 1999 96 5592 5597 10.1073/pnas.96.10.5592 10318929 PMC21905

[B99] Stewart JE Kawabe M Abdo Z Arie T Peever TL Contrasting Codon usage patterns and purifying selection at the mating locus in putatively asexual *Alternaria* fungal species PLoS ONE 2011 6 e20083 10.1371/journal.pone.0020083 21625561 PMC3098265

[B100] Roberts RG *Alternaria yaliinficiens* sp. nov. on Ya Li pear fruit: from interception to identification Plant Dis 2005 89 134 145 10.1094/PD-89-0134 30795215

[B101] Boykin LM Armstrong KF Kubatko L DeBarro P Species delimitation and global biosecurity Evol Bioinformatics 2012 8 1 37 10.4137/EBO.S8532 PMC3256992 22267902

[B102] Rossman AY Palm-Hernández ME Systematics of plant pathogenetic fungi: why it matters? Plant Dis 2008 92 1376 1386 10.1094/PDIS-92-10-1376 30769568

[B103] Elena K Alternaria brown spot of Minneola in Greece; evaluation of citrus species susceptibility Plant Pathol Eur J Plant Pathol 2006 115 259 262 10.1007/s10658-006-9005-8

[B104] Golmohammadi M Andrew M Peever TL Peres NA Timmer LW First report of brown spot of tangerine hybrid cultivars Minneola, page and fortune caused by *Alternaria alternata* in Iran Plant Pathol 2006 12 2005 2098

[B105] Marín JE Fernández H Peres NA Andrew M Peever TL Timmer LW First report of Alternaria brown spot of citrus caused by *Alternaria alternata* in Peru Plant Dis 2006 90 686 10.1094/PD-90-0686C 30781168

