# Supplementary material for: Discord between morphological and phylogenetic species boundaries: incomplete lineage sorting and recombination results in fuzzy species boundaries in an asexual fungal pathogen
Source: BMC Evol Biol. 2014 Mar 3;14:38. doi: 10.1186/1471-2148-14-38 (PMC4015827; doi:10.1186/1471-2148-14-38)
Supplement: Additional file 1: Table S1 — Isolates used in the study [103,105]. [file 1471-2148-14-38-S1.doc]

| Table S1. Isolates used in the study [103-105]. | | | | | | |
| --- | --- | --- | --- | --- | --- | --- |
| Country | Isolate # | Host | Tissue | Collector | Patho.^e^ | Haplotype / Lineage ID |
| Misiones, Argentina | Arg189 | Murcott tangor | Fruit | J. Agostini | nt | H10, 2 |
| Narara, Australia | AUS3 | Ladu mandarin | Fruit | P. Broadbent | + ^a^ | H7, 3 |
| Narara, Australia | AUS11 | Emperor mandarin | Fruit | P. Broadbent | + ^a^ | H7, 3 |
| Aguai, Brazil | AGMC13 | Murcott tangor | Twig | R. Reis and N. Peres | + | H11, 1 |
| Aguai, Brazil | AGMC18 | Murcott tangor | Twig | R. Reis and N. Peres | + | H11, 1 |
| Casa Branca, Brazil | CBMC5 | Murcott tangor | Leaf | R. Reis and N. Peres | + | H9, 2 |
| Pereira, Columbia | Co45016 | Minneola tangelo | Leaf | B. Castro | +^a^ | H9, 2 |
| Pereira, Columbia | Co10CEN | Minneola tangelo | Twig | B. Castro | +^a^ | H9, 2 |
| Florida, United States | FL-SH-22 | Minneola tangelo | Leaf | T.L. Peever | +^a^ | H9, 2 |
| Florida, United States | FL-SH-1 | Minneola tangelo | Leaf | T.L. Peever | +^a^ | H11, 1 |
| Florida, United States | FL-SH-4 | Minneola tangelo | Leaf | T.L. Peever | +^a^ | H9, 2 |
| Florida, United States | FL-SH-32 | Minneola tangelo | Leaf | T.L. Peever | +^a^ | H9, 2 |
| Greece | GrAL4 | Minneola tangelo | n/a | K. Elena | +^b^ | H3, 3 |
| Mazandaran, Iran | Ir1 | Page mandarin | Leaf | M. Golmohammadi | +^c^ | H1, 3 |
| Mazandaran, Iran | Ir2 | Page mandarin | Fruit | M. Golmohammadi | +^c^ | H2, 3 |
| Mazandaran, Iran | Ir4 | Minneola tangelo | Leaf | M. Golmohammadi | +^c^ | H1, 3 |
| Yedidya, Israel | Is45002 | Minneola tangelo | Leaf | Z. Solel | +^a^ | H8, 1 |
| Hadassim, Israel | Is45006 | Minneola tangelo | Leaf | Z. Solel | +^a^ | H3, 3 |
| Hadassim, Israel | Is45008 | Minneola tangelo | Leaf | Z. Solel | +^a^ | H12,1 |
| Mayan Zvi, Israel | Is45010 | Minneola tangelo | Leaf | Z. Solel | +^a^ | H11, 1 |
| Mayan Zvi, Israel | Is45011 | Minneola tangelo | Leaf | Z. Solel | +^a^ | H3, 3 |
| Mayan Zvi, Israel | Is45013 | Minneola tangelo | Leaf | Z. Solel | +^a^ | H3, 3 |
| Calabria, Italy | It10 | Fortune tangerine | Fruit | P. Bella | nt | H6, 3 |
| Calabria, Italy | It17 | Fortune tangerine | Leaf | P. Bella | nt | H7, 3 |
| Calabria, Italy | It36 | Fortune tangerine | Leaf | P. Bella | nt | H7,3 |
| Calabria, Italy | It61 | Fortune tangerine | leaf | P. Bella | nt | H8, 1 |
| Satipo, Peru | Pe2 | Minneola tangelo | Leaf | N. Peres | +^d^ | H3, 3 |
| Satipo, Peru | Pe9 | Minneola tangelo | Leaf | N. Peres | +^d^ | H4, 3 |
| Satipo, Peru | Pe12 | Minneola tangelo | Leaf | N. Peres | +^d^ | H5, 3 |
| Satipo, Peru | Pe17 | Minneola tangelo | Leaf | N. Peres | +^d^ | H3, 3 |
| Tzaneen, South Africa | SA45057 | Minneola tangelo | Fruit | S.H. Swart | +^a^ | H3, 3 |
| Castellón, Spain | SP001 | Fortune tangerine | Leaf | A. Vicent | nt | H11,1 |
| Sihmurat, Turkey | Tu44160 | Minneola tangelo | Leaf | Y. Canihos | +^a^ | H3, 3 |
| Solakli, Turkey | Tu44162 | Minneola tangelo | Leaf | Y. Canihos | +^a^ | H13, 3 |
| ^a^ Peever et al. 2002. Phytopathology. 92: 794-802.  ^b^ Elena, K. 2006. European Journal of Plant Pathology. 115: 259-262.  ^c^ Golmohammadi et al. 2006. Plant Pathology. 12: 2005-98.  ^d^ Marín et al. 2006. Plant Disease. 90: 686.  ^e^ Pathogenicity tested for each isolates, +, pathogenic; nt, not tested  ^f^ Lineage designation based on the *endo-polygalacturonase* phylogeny | | | | | | |
